# Advances in the understanding and treatment of Cutaneous T-cell Lymphoma

**DOI:** 10.3389/fonc.2022.1043254

**Published:** 2022-11-24

**Authors:** Farrah S. Bakr, Sean J. Whittaker

**Affiliations:** St. John’s Institute of Dermatology, School of Basic and Biomedical Sciences, Kings College London, London, United Kingdom

**Keywords:** cutaneous T cell lymphoma (CTCL), dermatology, pathway targeted interventions, genomics, cutaneous lymphoma

## Abstract

Cutaneous T-cell lymphomas (CTCL) are a heterogeneous group of non-Hodgkin’s lymphomas (NHL) characterised by the clonal proliferation of malignant, skin homing T-cells. Recent advances have been made in understanding the molecular pathogenesis of CTCL. Multiple deep sequencing studies have revealed a complex genomic landscape with large numbers of novel single nucleotide variants (SNVs) and copy number variations (CNVs). Commonly perturbed genes include those involved in T-cell receptor signalling, T-cell proliferation, differentiation and survival, epigenetic regulators as well as genes involved in genome maintenance and DNA repair. In addition, studies in CTCL have identified a dominant UV mutational signature in contrast to systemic T-cell lymphomas and this likely contributes to the high tumour mutational burden. As current treatment options for advanced stages of CTCL are associated with short-lived responses, targeting these deregulated pathways could provide novel therapeutic approaches for patients. In this review article we summarise the key pathways disrupted in CTCL and discuss the potential therapeutic implications of these findings.

## Introduction

Cutaneous T-cell lymphomas are a heterogeneous group of Non-Hodgkin’s lymphomas of which Mycosis Fungoides (MF) is responsible for almost 50% of all primary cutaneous lymphomas (1). They display wide variation in relation to their clinical, histopathological, immunophenotypic and underlying biologic features (1, 2). CTCLs are amongst a wider group of mature T-cell malignancies of which the more common subtypes are Peripheral T-cell lymphoma, Not Otherwise Specified (PTCL nos) and systemic anaplastic large cell lymphoma (ALCL). A rare HTLV-1 driven subtype of T-cell lymphoma known as Adult T-Cell Leukaemia/Lymphoma (ATLL) shares several phenotypic and genetic features with Sézary syndrome (SS) (3). Though MF/SS are the focus of this review, we will compare and contrast genomic abnormalities of MF/SS with other CTCL variants and systemic T-cell lymphomas and highlight potential novel therapeutic strategies.

## Genomic landscape of Mycosis fungoides and Sézary syndrome

In recent years, multiple high-throughput next-generation sequencing (NGS) studies have identified a complex genomic landscape in MF/SS, including high rates of somatic non-synonymous variants (SNVs) and copy number variants (CNVs) (4–13). The majority of studies have utilised whole exome sequencing (WES) of peripheral blood samples enriched for CD4+ leukemic T-cells from SS patients (4–9, 12, 13). Fewer samples from advanced stage MF have been analysed (n=56) by WES or whole genome sequencing (WGS) platforms (4, 5, 7, 10–12). An independent analysis of genomic data published prior to 2017 encompassing 220 CTCL cases (186 SS; 25 MF; 9 CTCL NOS) has highlighted at least 55 putative driver genes affecting multiple signalling pathways (13). Interestingly, there is significant overlap between MF and SS in the pathways affected. Commonly perturbed genes include those involved in TCR signalling pathways (*PLCG1; CARD11; CD28; RLTPR*) and those that selectively up-regulate the NFkB pathway (13). Other disrupted pathways include the DNA damage response (*TP53; POT1; ATM; BRAC1-2*), chromatin modification (*ARID1A; TRRAP; DNMT3A; TET2*) and JAK STAT signalling (*STAT5B; JAK3*). Critically the aforementioned gene variants have been functionally validated confirming their driver gene status (4–8, 12–14). A summary of the pathways and their associated gene mutations is provided in **Table 1**.

**Table 1:** Major aberrant pathways and associated emerging systemic therapies for advanced MF/SS.

Pathway	Gene	Systemic agent class	Agent name	Mechanism of action	References
TCR Signalling	PLCG1	PLCG1 inhibitor	In development	Inhibition of PLCG1 leading to decreased TCR signalling	--
	CD28				
	CARD11				
	PRKCB/Q	PKCθ inhibitor	Sotrastaurin	Inhibition of STAT3, leading to decreased cell proliferation and apoptosis in pre-clinical studies.	Garcia-Diaz (15)
	RLTPR				
	PTPRN2				
JAK-STAT pathway	JAK1-3	JAK inhibitor	Ruxolitinib	JAK1/2 inhibitor. Dysregulated JAK-STAT pathway in CTCL leads to T-cell activation. Phase II trial showed 23% ORR.	Moskowitz (16)
	STAT3-5				
DNA Damage Repair	ATM	ATR inhibitor	VE-821/2 ETP-46464 AZD6738	Synthetic lethality in view of HR defects. Decreased cell viability in SS cells and increased sensitivity to phototherapy in CTCL cell lines in pre-clinical studies.	Biskup (17) Pinzaru (3)
	BRCA1/2				
	RAD50/51C				
	Chk2				
	POT1				
Chromatin Modification	TET2	HDAC inhibitor	Resminostat Vorinostat Romidepsin	HDACi modify acetylation sites in proteins leading to dysregulated gene transcription, cell cycle arrest and apoptosis. Resminostat phase II trial ongoing (RESMAIN NCT02953301). Vorinostat and Romidepsin are FDA and EMA approved.	Olsen (18) Whittaker (19)
	ARID1A/B				
	DNMT3A				
	SMARCB1				
	SETDB2				
	TRRAP				
	CREBBP				
	NCOR1				
	BCOR				
	CTCF				
	KMT2C-D				
Cell cycle	CDKN2A	--	--	--	--
	TP53				
NFkB pathway	TNFRSF1B	Proteasome inhibitor	Bortezomib	Inhibits the degradation of the nuclear factor kappa B (NFκB) inhibitor IκBα. Phase II trial showed 67% ORR.	Zinzani (20)
	NFKB2				
	PRKCB				
	TNFAIP3				
	IRF4				
	CSNK1A1				
T-cell migration	CCR4	CCR4 antibody	Mogalizumab	Increased CCR4 expression seen in CTCL. Phase III trial (MAVORIC) of CCR4 inhibitor leads to increased PFS and OR vs. Vorinostat in SS.	Kim (21)
MAPK pathway	NF1	--	--	--	--
	RAS				
	BRAF				
	MAP2KI				
	MAPK1				
PI3K pathway	VAV1	Phosphatidylinositol 3-kinase (P13K) inhibitor	Duvelisib	PI3K-δ and PI3K-γ active in leucocytes and important for modulating immune response and tumour microenvironment. Phase I trial showed 32% ORR.	Horwitz (22)
	ARHGEF3				
	RHOA				
PD-1 pathway	PRKCB	Anti-PD1/PD-L1 inhibitors	Pembrolizumab Nivolumab	Increased PD-1 expression in CTCL. Inhibition enhances cytotoxic T-cell killing. Phase II trial showed 38% ORR.	Khodadoust (23)
	PD-L1				
	PD-L2				

A summary of the main pathways harbouring putative driver gene mutations or copy number changes in MF/SS. Novel systemic therapies targeting these pathways currently under investigation are also highlighted. FDA, US Food and Drug Administration; EMA, European Medicines Agency.

There is limited data available on chromosomal rearrangements in view of the small WGS datasets from advanced MF samples. However, complex patterns of chromosomal rearrangements and translocations with no recurrent balanced translocations have been frequently identified with specific gains (17q, 8q) and losses (10q, 17p) (24).

### T-cell signalling and differentiation

Mutations in *PLCG1* and *CARD11* are two of the most frequently observed in MF/SS. They appear to be mutually exclusive and occur in almost 30% of SS cases (13). These gain of function mutations increase downstream T-cell signalling specifically through enhanced NFkB, NFAT and AP1 transcriptional activity (12, 14). These transcription factors regulate the expression of genes involved in cell proliferation, survival and differentiation. Crucially, there is evidence that many of these variants induce constitutive activation of downstream T-cell signalling without T-cell stimulation (12, 14). Furthermore, CTLA4-CD28 and ICOS-CD28 gene fusions enhance CD28 dependent T-cell signalling, and *RLTPR* variants activate the NFkB pathway thereby increasing downstream TCR signalling (7, 13). The high prevalence of these of NFkB pathway gene variants in CTCL supported by functional data indicates that there is a critical selection pressure for activation of the NFkB pathway in the transformation of mature T-cells.

*PLCG1* mutations have also been detected in other mature T-cell malignancies, notably HTLV-1 associated ATLL (25), PTCL(NOS) (26), hepato-splenic T-cell lymphomas (27) and AITL (28) with the *PLCG1* p.S345F and R48W variants being two of the most frequently reported. In addition, mutations of the *JAK-STAT, CD28, VAV1, DNMT3A* and *TET2* genes are also reported in other mature T-cell malignancies (4–9, 25–28).

Enhanced T-cell activation *via* the T-cell receptor and NFkB pathway, leads to downstream activation of multiple pathways including the Janus tyrosine kinase (JAK) Signal transducers and activators of transcription (STAT) pathway. These proteins have a multitude of functions including TĤ cell proliferation and differentiation, as well as gene regulation and epigenetic modification. Unlike the STAT proteins, gain of function mutations in the JAK proteins are infrequently observed. However, copy number gains of both *STAT3* and *STAT5B* are common and associated with constitutive expression of these key transcription factors (29, 30).

### Epigenetic modification

Epigenetic changes include DNA and histone modifications which affect gene transcription and regulate cell differentiation. Furthermore, epigenetic modification is critical for sustaining the transcriptional memory for T-cells allowing rapid transcription of inducible genes upon activation (31, 32). Global hypomethylation is a consistent feature of malignancy and contributes to genomic instability but DNA methylation of gene promoters can lead to gene silencing. Crucially these changes are observed in MF/SS with evidence for hypomethylation of 7.8% of CpG sites in SS, and hypermethylation of 3.2% of CpG sites, specifically in the proximal region of promoters (33). There is extensive evidence that promoter hypermethylation leads to the silencing of specific tumour suppressor genes in MF/SS including those involved in cell cycle regulation (*CDKN2A/2B*) (34), DNA repair (*MLH1* and *MGMT*) *(* 35), apoptosis (*FAS*) (36) and JAK-STAT signalling (*SHP-1*) (37). Methylation of cytosine residues to 5-methylcytosine is mediated by DNA methyltransferases (DNMTs) and gain of function mutations of *DNMT3A* have been frequently identified in haematologic malignancies including MF/SS (4, 5, 8, 9). A second type of DNA methylation involves 5-hydroxymethylation of cytosine which is mediated by ten-eleven translocation 1-3 enzymes (TET1-3) but, in contrast to 5-methylcytosine, this is associated with enhanced gene expression (38). Loss of function T*ET2* mutations are well documented in SS (5, 7–9, 30). In SS, there is also mutational selection pressure for genes involved in other epigenetic modifications including *IDH* encoding isocitrate dehydrogenases which inhibit TET proteins, *ARID1A/1B* which affect chromatin modelling and *MLL* genes which mediate histone methyltransferases (4, 8, 9, 30). Large epigenomic studies in MF/SS have shown that the methylation pattern of leukemic T-cells in SS can be similar to that of regulatory T-cells and that there is almost universal activation of NFkB (39). Recent data suggest that hypermethylation of the hTERT promoter in MF/SS may be associated with telomerase activation (40).

MicroRNA (miRs), one of a group of non-coding RNA transcripts, are key post-transcriptional regulators of mRNA and are known to affect both the stability and translation of mRNA. In MF/SS, miR dysregulation has been observed with aberrant expression linked to abnormal DNA methylation of miR promoters as well as copy number changes (41).In addition, constitutive activation of STAT3/5 has been shown to enhance miR-155 and miR-21 expression leading to increased apoptosis resistance and Th2 proliferation (41).

### DNA damage response pathways and telomere instability

The DNA damage response (DDR) consists of numerous complex and inter-dependent signalling pathways which either maintain cell viability by repair of DNA or direct the damaged cell to undergo senescence or programmed cell death. Inevitably this complex process is closely linked with pathways regulating the cell cycle, chromatin remodelling and apoptosis (42). Previous cytogenetic and array CGH studies in MF/SS identified complex structural and numerical chromosomal abnormalities (24). More recent WGS and WES studies have confirmed a high degree of genomic instability with over 60 gene aberrations reported across all 5 DDR pathways (5, 8–10). One study of 101 SS samples identified SNVs and/or CNVs affecting genes involved in DNA repair and telomere maintenance in over 50% of cases. Notably, SNVs and CNVs affecting genes involved in homologous recombination such as *RAD51C, BRAC2* and *POLD1*, are also detected in MF/SS (9). *TP53* is the most commonly mutated gene in CTCL with loss of function SNVs and deletions which lead to a significant detrimental effect on the DNA damage response and telomere stability.

Telomere dysregulation is a recognised feature of MF/SS where shortened telomeres have been observed (43, 44). Furthermore, mutations in *POT1*, encoding a telomere binding protein are also frequently detected in MF/SS and ATLL (4, 5, 8–11, 25, 30, 45) and studies suggest that these loss of function variants likely contribute to telomere dysfunction by abolishing telomere binding and inducing DNA damage at telomeres in the form of telomere induced foci (TIFs) (3, 46–51). Cell cycle dysregulation is a major contributor to tumorigenesis in MF/SS. Mutation or deletions have been reported in several cell cycle checkpoint and tumour suppressor genes including CDKN1, CDKN2A, CDKN2B, ATM, ATR, TP53, RB1 and PTEN (4, 5, 8–10). Loss of function ATM mutations have been reported in several T-cell lymphomas (25, 26, 52–60) including MF/SS (9–11) and ATR mutations are also observed in MF/SS and NK T-cell lymphoma (5, 8, 10, 11, 61). In view of the key role of these kinases in the DDR and in cell cycle regulation, pathway disruption either by gene mutation or as a result of telomere dysfunction is likely to contribute to the genomic instability seen in MF/SS.

## CTCL evolution

As the vast majority of studies have been conducted in samples from advanced stages of MF or leukaemic SS samples, until recently there has been little insight into the driver events in early-stage disease. In solid malignancies, the use of mathematical modelling of WGS/WES data has deepened understanding of the evolution of genomic events and the impact of intra-tumour heterogeneity on therapeutic response (62). The use of paired plaques and tumours from MF patients has demonstrated that sub-clonal evolution is a feature of MF and is linked to disease progression, however, as yet these preliminary studies have failed to identify a common series of genomic events in early stages of disease (11).

## CTCL causation

A series of mutational signatures have been identified which are linked to a combination of intrinsic and extrinsic mutagens, and have been associated with specific malignancies (63, 64). The presence of these mutational signatures in clonally expanded cell populations is determined by assessing the six substitution types and their 5’ and 3’ nucleotide context giving 96 different trinucleotide mutation types. A recent meta-analysis of whole exome sequencing (WES) data from 403 patients across several T-cell NHL subtypes, including 6 MF/SS studies has revealed that the UV signature (signature 7) was exclusively present in MF/SS. It accounted for the mutational burden in 52% of MF and 23% of SS cases (65). In addition, these C>T/CC>TT mutations at dipyrimidine sites had a significant bias towards the untranscribed strand which is a feature of transcription-coupled nucleotide excision repair associated with UV-induced mutations (66). Crucially, the presence of this signature (7.5-88% of the overall SNVs) in CD4+ cells isolated from the blood of SS patients suggests that these malignant T-cells either circulate freely from the skin to the blood compartment or develop from skin resident memory T-cells. A significant proportion of patients analysed (41%) were treatment naïve and the detection of a mutational signature is dependent on the presence of a clonal population suggesting that the malignant T-cell in MF/SS accumulates UV associated mutations before transformation and clonal expansion.

Similar to other malignancies linked to exogenous mutagens (e.g. non-melanoma and melanoma skin cancers and smoking-associated lung cancers), MF/SS exhibit a very high mutational load, and are unique amongst other types of T-cell lymphoma which carry a much lower mutational burden. These data confirm the significant contribution of environmental UV exposure to the mutational burden in MF and SS and UV is a likely causal factor in the transformation of T-cells that are either circulating through or resident in skin.

## Other CTCL variants

### Primary cutaneous CD30+ lymphoproliferative disorders (pcALCL/LYP)

Primary cutaneous CD30+ anaplastic large cell lymphoma (pcALCL) has similar genomic alterations to its systemic counterpart albeit at much lower frequency. Most fail to express anaplastic lymphoma kinase (ALK), but have an excellent prognosis (67). In a small proportion of patients mutations of *JAK1* and/or *STAT3* and *NPM1-TYK2* gene fusions been reported in pcCD30+ALCL (68).

Chromosomal rearrangements involving the *DUSP22-IRF4* (MUM1) locus on 6p25.3 have also been identified in both pcCD30+ ALCL (25%) and less commonly (5%) in lymphomatoid papulosis (LYP) (69), but MUM1 expression is not specific for this rearrangement.

#### Subcutaneous panniculitis-like T-cell lymphoma (SPTCL)

The majority of cases harbour one of two homozygous loss of function germline *HAVCR2* variants (pTyr82Cys and p.Ile97Met) observed in Polynesian/East Asian and European origin respectively (70). *HAVCR2* encodes T-cell immunoglobulin mucin 3 (TIM-3) is expressed by CD8+ T-cells and NK cells and regulates peripheral tolerance, innate immunity and inflammatory responses. Somatic variants have also been detected in genes involved in epigenetic modification (*TET2, ARID1B*), the PI3K/AKT/mTOR and JAK-STAT pathways (71).

#### Primary cutaneous γδ T-cell lymphomas

Similar to MF/SS, mutations in the JAK/STAT, MAPK, MYC and chromatin modification pathways have been detected, but interestingly, TCR-CD28 signalling pathway mutations have also been reported (72). In addition, panniculitic Vδ2 T-cell lymphomas do not show germline mutations of *HAVCR2* as seen in a majority of αβ SPTCL patients (72).

## Prognostic biomarkers

The prognosis for patients with MF/SS is variable even amongst patients with the same stage of disease. This has been partly addressed by the proposal of clinical prognostic models such as the CLIPi (73, 74) which is currently the subject of a multi-centre prospective study. In view of the genomic heterogeneity observed in MF/SS, it is likely that a more accurate prognostic model will require analysis of genetic clusters which may include a combination of gene mutations including SNVs and CNVs. There is some evidence that specific genomic clusters can be defined in SS (75), however, there has been no clinical correlation with patient outcomes to date. Furthermore, the paucity of genomic data in MF highlights the need for further NGS studies, particularly in early stage disease.

## Therapeutic implications

In view of the genomic heterogeneity in MF/SS reflecting the underlying high rate of UV signature mutations, a single targeted treatment option is unlikely to be effective. However, a deeper understanding of the genomic landscape could provide insight into potential therapeutic approaches especially in early stage disease (76). Stratification of individual patients according to mutational profile/deregulated pathway could allow the use of existing treatments such as Ipilimumab (*CD28-CTLA4* fusion), Ruxolitinib and Tofacitinib (*JAK* mutations or *JAK2 fusions* as detected in rare aggressive cytotoxic CTCL variants) (77, 78) and Bortezomib (NFkB pathway) (79). Patients with abnormalities of epigenetic regulation such as *DNMT3A* and *TET2* could be selected for treatment with demethylating agents such as 5-Azactidine and/or HDACi, whilst those with *RHOA* mutations could be eligible for PI3K inhibitors (Duvelisib). However, an alternative is to consider a tumour agnostic approach and the genomic landscape of MF/SS including marked genomic instability suggest that targeting the DDR pathway might be a productive strategy. A summary of emerging treatments targeting the various dysregulated pathways in MF/SS is provided in **Table 1**.

### Restoration of Th1/Th2 immune profile

In view of the immune dysregulation profile seen in advanced MF/SS with a diminished Th1 immune response (IFN-gamma, IL-12) and skewing towards a Th2 immune profile (increased IL-4, IL-5 and/or IL-13) (80), interferons (IFN-alpha and IFN-gamma) were amongst the first immunotherapies to be used. IFN-alpha has been shown to stimulate antitumour cytotoxicity by activating CD8+ T-cells and NK cells and restores the Th1/Th2 balance by reducing IL-4 and IL-5 production by malignant T-cells (81, 82). IFN-gamma acts similarly to IFN-alpha by activating CD8+ and NK cells and increasing Th1 cytokine profile (80). IFN-alpha is EMA approved and effective in early stage patients who are refractory to skin-directed therapies (83), in which case it can be combined with phototherapy (84). Several clinical trials have highlighted the potential use of recombinant IL-12 as a novel immunotherapy with encouraging results (85–87).

### Targeted therapies

There are currently several trials underway targeting checkpoint molecules (88, 89). Mogamulizumab, an anti-CCR4 monoclonal antibody has recently been approved by FDA and EMA following a phase III clinical trial which showed increased progression free survival (PFS) compared with the HDAC inhibitor, Vorinostat (21). Recently, it has emerged that Mogamulizumab also contributes to efficient immune restoration involving CD8+ as well as stem and memory CD4+ cells (90).

Pembrolizumab and Nivolumab inhibit the PD-1 receptor which enhances cytotoxic T-cell killing and have shown clinical responses in phase I and II trials (23, 91, 92). Whilst the high mutational burden of MF/SS would suggest that immunotherapies should be successful, the modest responses from these phase II trials (93) highlight the challenges of using a PD-1 inhibitor on a T-cell lymphoma which can express PD-1. Specifically there are two scenarios: PD-1 expression might reflect underlying gain of function mutations which would benefit from inhibition and there is emerging data suggesting that PD-1 mutations can be detected in some MF/SS patients (94), or PD-1 inhibition might reverse the tumour cell exhaustion leading to activation and proliferation of malignant T-cells. Whilst this second scenario has not yet been seen in MF/SS patients receiving PD-1 inhibitors, a phase II trial of Nivomumab in ATLL was discontinued because of rapid disease progression (95).

Targeting of CD47 with intralesional or intravenous TTI-621 has been used in patients with relapsed or refractory MF/SS in recent phase I trials with encouraging results (96, 97). Recent reports have shown that the CD39-CD73-adenosine pathway generates an immunosuppressive tumour microenvironment in SS and this provides a potential option to use emerging novel therapeutic approaches to target this pathway possibly in combination with checkpoint inhibitors or Mogamulizumab (98, 99).

### DNA damage repair pathway

**Figure 1:**
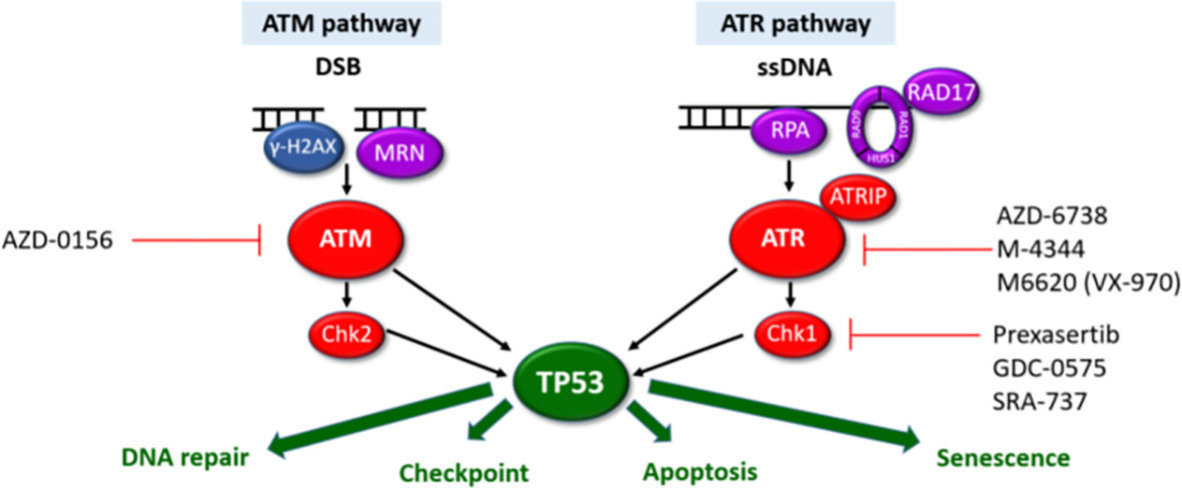
ATM and ATR DDR pathways. The ATM and ATR pathways respond to different types of DNA damage with separate sensors (purple), mediators (blue) and transducers (red). Synthetic lethality can be exploited for cells with aberrant DDR pathways. Cells harbouring LOF mutations in one pathway have increased reliance on the other pathway for DNA damage repair. Inhibition of the intact pathway prevents p53-mediated DNA repair, cell cycle arrest and apoptosis. This results in accumulation of unrepaired DNA damage, mitotic catastrophe triggering p53-independent cell death. Pharmacological agents targeting components of the ATM/ATR pathways currently in clinical development are highlighted.

Perhaps the most interesting option would be tumour agnostic therapies targeting the DNA damage response (DDR) pathways which show considerable promise in various solid malignancies often as maintenance therapies after platinum-based chemo regimens (42). The DDR pathways are a vast network of over 450 proteins. These therapeutic approaches build on the success of PARP inhibitors inducing synthetic lethality in homologous recombination (HR) deficient malignancies (due to BRCA1 or BRCA2 loss). In view of the somatic mutations or deletions of HR genes (including *ATM, BRCA1, BRCA2, Chk2, RAD50, RAD51C)*, these could be repositioned for use in MF/SS patients (**Figure 1**). Several ATR inhibitors are in early clinical development for use in both solid and haematological malignancies (100, 101). There is increasing evidence that ATR inhibition may be a potential therapeutic target in MF/SS. Small molecule inhibitors of ATR and Chk1 (VE-821/2 and Chir-124) have been shown to sensitise MF/SS cell lines to phototherapy by inducing apoptosis (17). In addition, cells overexpressing POT1 mutants (p.F62V and p.K90E) treated with an ATR inhibitor (ETP-46464) resulted in significant abrogation of TIFs (3). ATM inhibition has also been shown to overcome HDAC inhibitor resistance in both B and T-cell derived lymphomas including MF/SS, providing a rationale for combination therapy (102). Acting immediately downstream of ATR, targeting of Chk1 is effective in MYC-driven tumours including B-cell lymphomas owing to MYC-induced replication stress (103). Amplification of the MYC oncogene is one of the most commonly observed aberrations in MF/SS (4) which could increase replication stress in these cells and hence increase their sensitivity to ATR and/or Chk1 inhibitors. Furthermore, Chk1 inhibitors synergise with a number of therapeutic agents to induce cell death in MF/SS including the proteasome inhibitor, Ixazomib (104) and phototherapy (17).

## Conclusions

Despite the wide range of treatment options currently available for MF/SS, therapeutic responses are invariably in the region of 30% and usually short lived (105), highlighting the need for a better understanding of the pathogenic mechanisms which would enable the development of more targeted therapies. A deeper understanding of dysregulated pathways and immunology in recent years has facilitated the development of several novel drugs currently in clinical trials. In addition to modulating pathways such as the JAK-STAT and NFĸB pathways and immune checkpoints, targeting genomic instability and the DDR present an exciting novel treatment approach for T-cell malignancies such as MF/SS.

## Author contributions

FB and SW planned and wrote the manuscript. Figure and table was generated by FB. All authors contributed to the article and approved the submitted version.

## Funding

Open access fees are provided by King’s College London *via* the grant code: ST461.

## Conflict of interest

The authors declare that the research was conducted in the absence of any commercial or financial relationships that could be construed as a potential conflict of interest.

The reviewer VG declared a past collaboration with the author SW to the handling editor.

## Publisher’s note

All claims expressed in this article are solely those of the authors and do not necessarily represent those of their affiliated organizations, or those of the publisher, the editors and the reviewers. Any product that may be evaluated in this article, or claim that may be made by its manufacturer, is not guaranteed or endorsed by the publisher.
